# Trachoma Prevalence in Al Rahad Locality, Sudan: Evaluating *Chlamydia trachomatis* Infection Prevalence as a Complementary Programmatic Indicator

**DOI:** 10.4269/ajtmh.26-0096

**Published:** 2026-05-05

**Authors:** Scott D. Nash, Emmanuel A. Ackah, Balgesa E. Elshafie, Zeinab Abdalla, Sara Lavinia Brair, Tania A. Gonzalez, Charles A. Rivers, Barbara Van Der Pol, E. Kelly Callahan, Angelia M. Sanders

**Affiliations:** ^1^ The Carter Center, Atlanta, Georgia, USA;; ^2^ Sudan Federal Ministry of Health, Khartoum, Sudan;; ^3^ The Carter Center, Khartoum, Sudan;; ^4^ Heersink School of Medicine, University of Alabama at Birmingham, Birmingham, Alabama, USA

## Abstract

Trachoma remains endemic in Sudan. A 2009 baseline survey in Al Rahad locality revealed a trachomatous inflammation-follicular (TF) prevalence of 7.1%, prompting azithromycin mass drug administration (MDA). After three MDA rounds (2015–2017), a trachoma impact survey was conducted in 2017. This study’s aim was to estimate the prevalence of clinical signs and ocular *Chlamydia trachomatis* infection identified with DNA testing. A cross-sectional, cluster-random sampling design was used, and certified graders assessed participants for clinical signs and swabbed children for *C. trachomatis*. The TF prevalence was 6.3% (95% CI: 4.0–10.0), and *C. trachomatis* prevalence was 0.8% (95% CI: 0.2–3.1). *Chlamydia trachomatis* infection was clustered centrally in the locality within communities with high TF prevalence. Despite MDA interventions, TF remained above the established 5% elimination threshold, and *C. trachomatis* infection was observed. Complementary indicators, such as *C. trachomatis* infection, should be considered in trachoma programs as they aid in better understanding trachoma endemicity.

## INTRODUCTION

Trachoma, a neglected tropical disease caused by repeated infection with ocular *Chlamydia trachomatis*, remains a public health problem in parts of Africa, including Sudan. Sudan has made significant progress toward elimination, completing nearly all baseline surveys, including in the conflict-affected Darfur region and within refugee camps.^1^^–^^4^ These surveys have shaped intervention strategies and informed programmatic progress. Where the surgery, antibiotics, facial cleanliness, and environmental improvement strategy has been implemented, measurable success has been observed.^5^^,^^6^

Although the clinical sign trachomatous inflammation-follicular (TF) remains the primary programmatic indicator for monitoring trachoma, it may overestimate *C. trachomatis* infection, particularly after the start of mass drug administration (MDA) interventions.^7^^,^^8^ Increasingly, trachoma programs are incorporating complementary indicators, like *C. trachomatis* infection testing, into surveys to enhance the understanding of underlying infection dynamics in endemic areas. Having both TF and *C. trachomatis* prevalence may be particularly helpful when a district nears the <5% TF elimination threshold.

Al Rahad locality (district) in Gedarif state was historically endemic for trachoma.^1^ After a 2009 baseline survey, three annual rounds of MDA were implemented. A 2017 trachoma impact survey (TIS) assessed the prevalence of both clinical signs and ocular *C. trachomatis* infection among children using DNA-based diagnostics. These findings provide insight into trachoma endemicity in Al Rahad and inform the global program on the utility of *C. trachomatis* testing in low-endemic settings.

## MATERIALS AND METHODS

The survey methodology was approved by the Sudan Federal Ministry of Health’s National Research Ethics Review Committee and Emory University under protocol 079-2006. Given the high illiteracy rate in the population, institutional review board approval was granted for oral consent or assent for older children, which was recorded electronically.

To estimate TF prevalence among children ages 1 to 9 years old, a prevalence of 3%, ±2% precision, and a design effect of 3.0 were assumed. This yielded a required sample size of 837 children. Adjusting for an anticipated nonresponse rate of 20% increased the target to 1,004 children. Assuming 4.7 individuals per household and assuming that 35% of the population is 1 to 9 years old, approximately 611 households were needed. Given that 25 households could be surveyed in 1 day, a sample of 25 clusters, each with 25 households, was selected. A two-stage cluster sampling design was used. First, 25 clusters were selected using probability proportional to the estimated size from a complete list of villages in the locality. Second, within each selected cluster, five segments of approximately five households were randomly selected using existing household lists maintained at village offices. All households in the selected segment were visited.

Trachoma graders underwent standardized training, including both classroom and practical components. Certification required passing intergrader reliability examinations (slide and field tests) with a kappa score (κ) >0.7 for TF compared with a certified master grader (BEE). Graders were further trained on conjunctival swabbing using standard procedures.^8^ Recorders participated in a 3-day training with classroom and field practice.

During the survey, heads of households were asked about their household’s access to water, sanitation, and hygiene. After household-level enumeration, all individuals ages 1 year old and older were examined for trachoma using the WHO simplified clinical signs.^9^ Children ages 1 to 9 years old also provided conjunctival swabs.

Swabs were collected from the left eye of each child using sterile polyester-tipped applicators. Graders wore new gloves for each participant and swabbed the upper tarsal conjunctiva three times with a 120° rotation between passes.^8^ Approximately one control “air swab” was collected in each village. Swabs were immediately placed into dry, sterile cryotubes and stored in cold boxes in the field. Samples were transported on ice packs to the Khartoum Public Health Laboratory for initial storage at −20°C. In 2022, they were then shipped on ice packs to the University of Alabama at Birmingham for DNA-based testing using the Roche Cobas^®^ (Mannheim, Germany) 6,800 CT/NG polymerase chain reaction assay.^10^ Samples were tested individually, and laboratory technicians were masked to the clinical status of the samples and whether a swab was a negative “air swab” or a participant swab.

The prevalences of clinical signs; *C. trachomatis* infection; and water, sanitation, and hygiene indicators were estimated using survey weights to account for the multistage sampling design. Robust 95% CIs were estimated using Taylor linearization with cluster- and household-level adjustment.^5^ Logistic regression analyses were conducted using the survey package in R v. 2023.06.0 + 421 (R Foundation, Vienna, Austria) accounting for clustering and weights to evaluate associations between age, sex, TF, and *C. trachomatis* infection. Maps were created using ArcGIS Pro v. 3.4.2 (ESRI, Redlands, CA).

## RESULTS

In December 2017, 514 households were surveyed, and 2,608 individuals were enumerated (Table 1). Among the 924 children ages 1 to 9 years enumerated, 860 (93.1%) were examined and swabbed. Household latrine access was 59.8% (95% CI: 44.8–73.1), and access to an improved water source was 35.9% (95% CI: 19.3–56.6).

**Table 1:** Demographic and household characteristics of survey participants in Al Rahad, Sudan in 2017

Characteristic	Total	Children (1–9 years old)	Adults (15+ years old)
Individual-level characteristics			
Total individuals	2,608	924 (35.4%)	1,684 (64.6%)
Sex			
Male	1,243 (47.7%)	475 (51.4%)	768 (45.6%)
Female	1,327 (52.3%)	441 (48.6%)	886 (54.4%)
Clean face, 1–9 years old	72.3% (95% CI: 60.2–81.8)	–	–
Household-level characteristics			
Total households	514	–	–
Latrine	59.8% (95% CI: 44.8–73.1)	–	–
Improved latrine	0.6% (95% CI: 0.1–2.7)	–	–
Water access (≤30 minutes)	89.8% (95% CI: 66.8–97.5)	–	–
Improved water source	35.9% (95% CI: 19.3–56.6)	–	–
Any adult education	86.2% (95% CI: 75.2–92.8)	–	–

Clean face indicates the absence of ocular and nasal secretions. Improved latrine indicates a sanitation facility designed to hygienically separate human excreta from human contact, including ventilated flush, flush, or pour-flush toilets and ventilated improved pit latrines. Improved water source indicates water from sources protected from outside contamination, such as a protected spring, hand pump/tube well, public piped water/standpipe, or private piped water into household.

**Figure 1.**
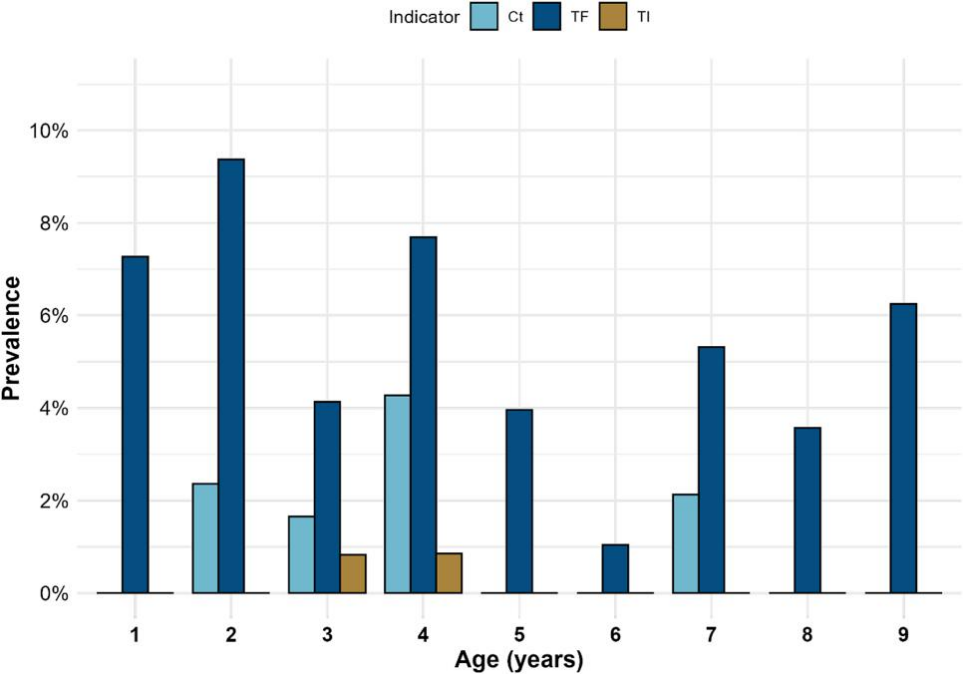
Prevalence of trachomatous inflammation-follicular (TF), trachomatous inflammation-intense (TI), and *Chlamydia trachomatis* (Ct) infection by age from 1 to 9 years old in Al Rahad, Sudan in 2017.

Trachomatous inflammation-follicular prevalence among children ages 1 to 9 years old was 6.3% (95% CI: 4.0–10.0), and trachomatous inflammation-intense prevalence was 0.2% (95% CI: 0.0–0.8). Trachomatous inflammation-follicular prevalence did not differ by age (*P* = 0.297) (Figure 1) or sex (*P* = 0.879). Among adults ages 15 years old and older, prevalence of trachomatous trichiasis (TT) unknown to the health system was 0.7% (95% CI: 0.3–1.7).

**Figure 2.**
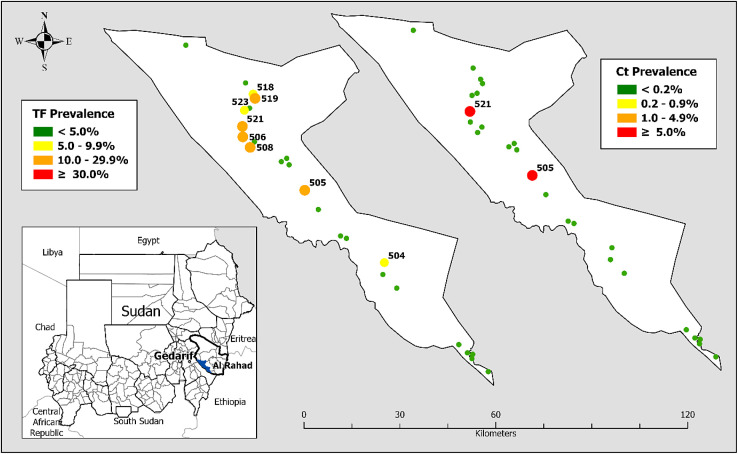
Prevalence of trachomatous inflammation-follicular (TF) and *Chlamydia trachomatis* (Ct) infection by cluster and geographic location in Al Rahad, Sudan in 2017.

*Chlamydia trachomatis* infection prevalence among children ages 1 to 9 years old was 0.8% (95% CI: 0.2–3.1), and prevalence among those ages 1 to 5 years old was 1.1% (95% CI: 0.3–4.4). All control swabs (*N* = 41) were negative. Of the 25 clusters, 13 clusters had at least one TF case; 2 of these 13 clusters also had confirmed *C. trachomatis* infections, with cluster-level *C. trachomatis* infection prevalences of 7.3% and 15.5% (Supplemental Figure 1). Both clusters with detectable infection were in the central part of the locality (Figure 2). Among the 47 children with TF, 6 (12.8%) were positive for *C. trachomatis* infection. In contrast, among the 809 children without TF, only 6 (0.75%) had *C. trachomatis* infection. Trachomatous inflammation-follicular was strongly associated with *C. trachomatis* infection, with an odds ratio (OR) of 19.6 (95% CI: 8.1–47.0). No significant associations were found between *C. trachomatis* infection and age (OR = 0.8, 95% CI: 0.6–1.1) or sex (OR = 1.1, 95% CI: 0.3–3.3).

## DISCUSSION

After three annual rounds of MDA, TF prevalence in Al Rahad remained above the elimination threshold. Further evidence of ongoing transmission was provided by the detection of *C. trachomatis* infection in some communities. Trachoma activities in Al Rahad locality began in 2009 when a baseline survey found a TF prevalence of 7.1%.^1^ A 2013 operational research survey targeting three sublocalities with suspected high prevalence revealed a TF prevalence of 29%, prompting three annual MDA rounds.^11^ Because of inconsistent findings across earlier surveys, the program integrated the collection of conjunctival swabbing for molecular testing of *C. trachomatis* infection into the 2017 TIS. This TIS supports baseline findings that Al Rahad likely had historically lower levels of endemicity; however, three rounds of MDA were insufficient to fully eliminate infection or reduce TF below the elimination threshold. Water, sanitation, and hygiene infrastructure was suboptimal, and TT cases remained to be operated. Although Sudan has made substantial progress, localities like Al Rahad, which are experiencing persistent trachoma, will require sustained attention to achieve elimination countrywide.^5^^,^^6^

Monitoring *C. trachomatis* infection alongside clinical signs is becoming more common within trachoma control programs. In Sudan, the collection of ocular swabs for *C. trachomatis* infection has been rare, typically limited to small samples or clinical settings.^12^^,^^13^ As a complementary indicator, *C. trachomatis* infection can be evaluated alongside TF to improve programmatic decision-making, particularly in districts near the elimination threshold where sampling variability may unduly affect estimates.^14^ In districts where the TF prevalence continues to persist above the TF threshold, it has now been recommended that programs consider including complementary indicators, such as *C. trachomatis* infection, into surveys.^15^ This study achieved a high response rate for swab collection, demonstrating feasibility in this setting. However, thresholds for *C. trachomatis* infection are still needed to help guide programmatic action. Here, the 1.1% *C. trachomatis* infection prevalence among children ages 1 to 5 years old, persistent TF ≥5%, and a strong association between *C. trachomatis* infection and TF at an individual level are evidence that this locality would benefit from continued interventions. As Al Rahad has been slow to respond to annual MDA, enhanced interventions, such as more frequent than annual (MFTA) MDA, may be warranted. Given the geographic clustering of *C. trachomatis* infection in central communities with high TF, the program may benefit from focusing MFTA MDA efforts within central subdistricts.

A limitation of this study was the delay between data collection and *C. trachomatis* testing. The shipment of samples was delayed by programmatic bottlenecks, the coronavirus disease 2019 pandemic, and the 2021 coup d’état in Sudan, with shipment not possible until 2022. This underscores the need for national laboratory capacity building if *C. trachomatis* infection is to become a programmatic indicator. Newer generation infection tests requiring less infrastructure could also facilitate wider implementation.^16^ Despite this limitation, this study used the Cobas^®^ 6800, a fully automated, high-throughput polymerase chain reaction system that can accurately and reliably detect *C. trachomatis* in clinical samples.^10^

## CONCLUSION

This study highlights the benefits of integrating *C. trachomatis* infection monitoring alongside TF in routine trachoma surveillance. Continuing to deliver high-quality MDA and hygiene promotion while incorporating more comprehensive program monitoring through molecular tools could significantly accelerate progress toward the elimination of trachoma as a public health problem in Sudan.

## Supplemental Materials

10.4269/ajtmh.26-0096 Supplemental Materials

## References

[1] Hassan A, , 2011. The prevalence of blinding trachoma in northern states of Sudan. PLoS Negl Trop Dis 5(5): e1027. 21655349 10.1371/journal.pntd.0001027 PMC3104955

[2] Sanders AM Abdalla Z Elshafie BE Nute AW Long EF Aziz N Weiss P Callahan EK Nash SD, 2019. Prevalence of trachoma within refugee camps serving South Sudanese refugees in White Nile State, Sudan: Results from population-based surveys. PLoS Negl Trop Dis 13(6): e0007491. 31194761 10.1371/journal.pntd.0007491 PMC6592575

[3] Sanders AM, , 2024. Serological responses to trachoma antigens prior to the start of mass drug administration: Results from population-based baseline surveys, North Darfur, Sudan. Am J Trop Med Hyg 111(Suppl 3): 49–57. 38507810 10.4269/ajtmh.23-0608 PMC11374501

[4] Elshafie BE Osman KH Macleod C Hassan A Bush S Dejene M Willis R Chu B Courtright P Solomon AW, 2016. The epidemiology of trachoma in Darfur states and Khartoum state, Sudan: Results of 32 population-based prevalence surveys. Ophthalmic Epidemiol 23(6): 381–391. 27841721 10.1080/09286586.2016.1243718 PMC5297557

[5] Sanders AM Abdalla Z Elshafie BE Elsanosi M Nute AW Aziz N Callahan EK Nash SD, 2019. Progress toward elimination of trachoma as a public health problem in seven localities in the Republic of Sudan: Results from population-based surveys. Am J Trop Med Hyg 101(6): 1296–1302. 31595874 10.4269/ajtmh.19-0530 PMC6896892

[6] Elshafie BE, , 2023. Trachoma prevalence in four localities of Darfur region, Sudan, following one round of antibiotic mass drug administration. Ophthalmic Epidemiol 30(6): 571–579. 34423732 10.1080/09286586.2021.1953538 PMC10581671

[7] Ramadhani AM Derrick T Macleod D Holland MJ Burton MJ, 2016. The relationship between active trachoma and ocular *Chlamydia trachomatis* infection before and after mass antibiotic treatment. PLoS Negl Trop Dis 10(10): e0005080. 27783678 10.1371/journal.pntd.0005080 PMC5082620

[8] Nash SD, , 2018. Ocular *Chlamydia trachomatis* infection under the surgery, antibiotics, facial cleanliness, and environmental improvement strategy in Amhara, Ethiopia, 2011–2015. Clin Infect Dis 67(12): 1840–1846. 29741592 10.1093/cid/ciy377 PMC6260158

[9] Thylefors B Dawson CR Jones BR West SK Taylor HR, 1987. A simple system for the assessment of trachoma and its complications. Bull World Health Organ 65(4): 477–483. 3500800 PMC2491032

[10] Cherkaoui A Renzi G Mombelli M Jaton K Yerly S Vuilleumier N Schrenzel J, 2019. Comparison of analytical performances of the Roche Cobas 6800 CT/NG assay with the Abbott m2000 Real Time CT/NG assay for detecting *Chlamydia trachomatis* and *Neisseria gonorrhoeae*. J Med Microbiol 68(2): 197–200. 30605081 10.1099/jmm.0.000909

[11] The Carter Center, 2013. *Summary Proceedings: Fourteenth Annual Trachoma Program Review*. Available at: https://www.cartercenter.org/publications/?_search_term=summary%20proceedings&_program_type=trachoma. Accessed January 15, 2026.

[12] Ghasemian E, , 2018. Detection of *Chlamydiaceae* and *Chlamydia*-like organisms on the ocular surface of children and adults from a trachoma-endemic region. Sci Rep 8(1): 7432. 29743637 10.1038/s41598-018-23887-1 PMC5943520

[13] Ghasemian E Inic-Kanada A Collingro A Mejdoubi L Alchalabi H Keše D Elshafie BE Hammou J Barisani-Asenbauer T, 2021. Comparison of genovars and *Chlamydia trachomatis* infection loads in ocular samples from children in two distinct cohorts in Sudan and Morocco. PLoS Negl Trop Dis 15(8): e0009655. 34370735 10.1371/journal.pntd.0009655 PMC8376198

[14] Gallini JW, , 2022. Optimizing cluster survey designs for estimating trachomatous inflammation-follicular within trachoma control programs. Int J Infect Dis 116: 101–107. 34965463 10.1016/j.ijid.2021.12.355

[15] World Health Organization, 2022. *Informal Consultation on End-Game Challenges for Trachoma Elimination, Task Force for Global Health, Decatur, United States of America, 7–9 December 2021*. Available at: https://www.who.int/publications/i/item/9789240048089. Accessed April 15, 2026.

[16] Derrick TR, , 2020. DjinniChip: Evaluation of a novel molecular rapid diagnostic device for the detection of *Chlamydia trachomatis* in trachoma-endemic areas. Parasit Vectors 13(1): 533. 33109267 10.1186/s13071-020-04414-6 PMC7590679

