# Supplementary material for: Trachoma Prevalence in Al Rahad Locality, Sudan: Evaluating Chlamydia trachomatis Infection Prevalence as a Complementary Programmatic Indicator
Source: Am J Trop Med Hyg. 2026 May 5;115(1):40–3. doi: 10.4269/ajtmh.26-0096 (PMC13326856; doi:10.4269/ajtmh.26-0096)

Supplemental Materials

Supplemental Figure 1. Prevalence of trachomatous inflammation-follicular (TF) and *Chlamydia trachomatis* (*CT)* infection by cluster, Al Rahad, Sudan, 2017.

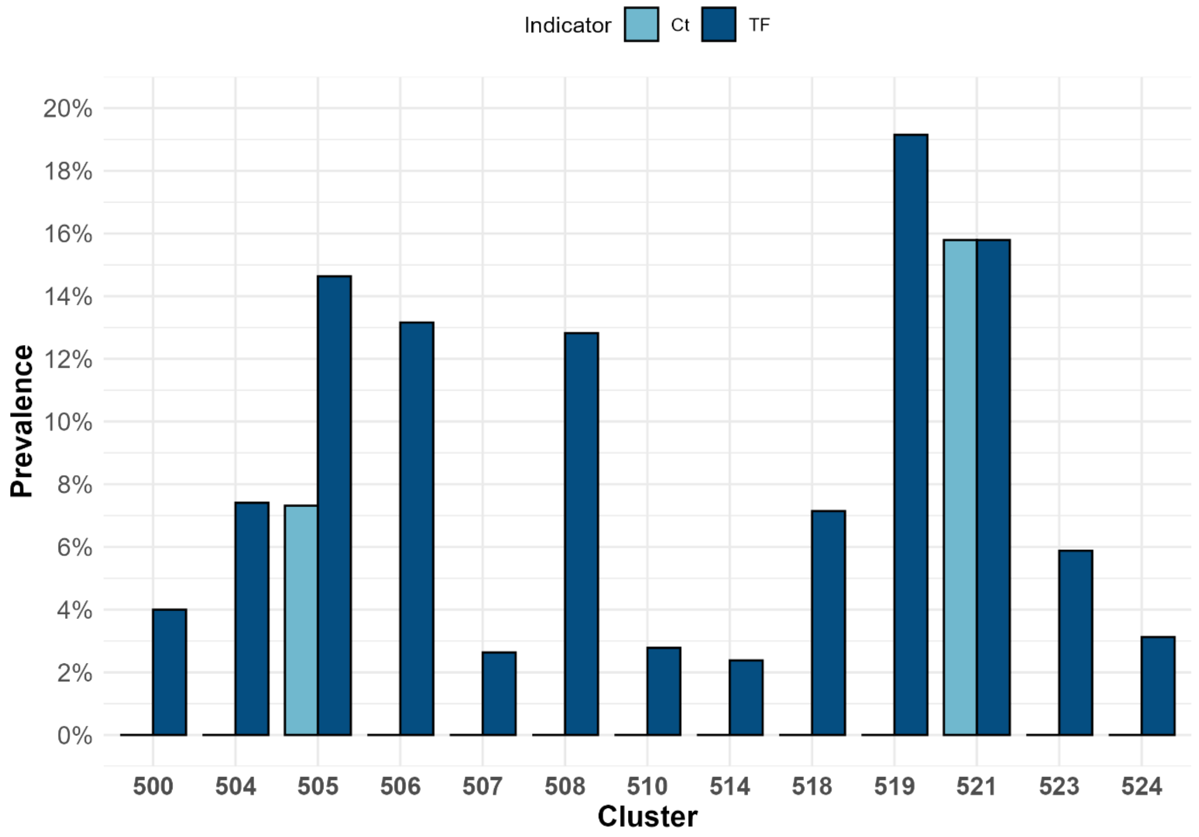

Supplement: Supplemental Materials [file tpmd260096.SD1.pdf]
